# Single cell analysis in native tissue: Quantification of the retinoid content of hepatic stellate cells

**DOI:** 10.1038/srep24155

**Published:** 2016-04-11

**Authors:** Kerstin Galler, Robert Pascal Requardt, Uwe Glaser, Robby Markwart, Thomas Bocklitz, Michael Bauer, Jürgen Popp, Ute Neugebauer

**Affiliations:** 1 Leibniz Institute of Photonic Technology, Jena, Germany; 2 Center for Sepsis Control and Care, Jena University Hospital, Germany; 3 Institute of Physical Chemistry and Abbe Center of Photonics, Friedrich Schiller University Jena, Germany; 4 Department of Anesthesiology and Intensive Care Medicine, Jena University Hospital, Germany; 5 InfectoGnostics Research Campus Jena, Center for Applied Research, Jena, Germany

## Abstract

Hepatic stellate cells (HSCs) are retinoid storing cells in the liver: The retinoid content of those cells changes depending on nutrition and stress level. There are also differences with regard to a HSC’s anatomical position in the liver. Up to now, retinoid levels were only accessible from bulk measurements of tissue homogenates or cell extracts. Unfortunately, they do not account for the intercellular variability. Herein, Raman spectroscopy relying on excitation by the minimally destructive wavelength 785 nm is introduced for the assessment of the retinoid state of single HSCs in freshly isolated, unprocessed murine liver lobes. A quantitative estimation of the cellular retinoid content is derived. Implications of the retinoid content on hepatic health state are reported. The Raman-based results are integrated with histological assessments of the tissue samples. This spectroscopic approach enables single cell analysis regarding an important cellular feature in unharmed tissue.

Single cell analysis is most relevant in the native cellular environment. This native habitat is the tissue association. Unfortunately, the low intrinsic contrast of individual cells against their surrounding often prevents such investigations or requires the application of non-physiological treatment, respectively. Assessments by Raman spectroscopy are feasible under physiologic conditions^1^,^2^. However, the chemical contrast of individual cells is, generally, rather low as well. Nevertheless, there are some cells storing specific molecules, the retinoids (or vitamin A). These retinol-derived substances give rise to a unique pattern in the Raman spectrum^2^. Such retinoid storing cells are mostly known from liver, but are also present in lung, kidney, intestine and pancreas^3^. The Raman spectroscopic detection of natively occurring retinoids in tissue samples has been reported for lung^4^ and liver^5^ tissue samples. Moreover, it was shown, that the hepatic stellate cell (HSC) which is the retinoid storing cell in the liver, is detectable in fresh whole liver samples by excitation of Raman scattering with 785 nm^2^. Thus, a technique for HSC identification with considerably less phototoxicity for the cells than the conventional excitation of retinoid auto-fluorescence at around 330 nm^6^ is available. However, a Raman spectrum comprises more information than only the occurrence or absence of a certain vibrational band.

Herein, the quantitative assessment of the retinoid content of individual HSCs in native liver is reported. Furthermore, first implications derivable from the hepatic retinoid state for the hepatic state of health are illustrated.

## Results and Discussion

### Retinoid calibration curve

**Figure 1:**
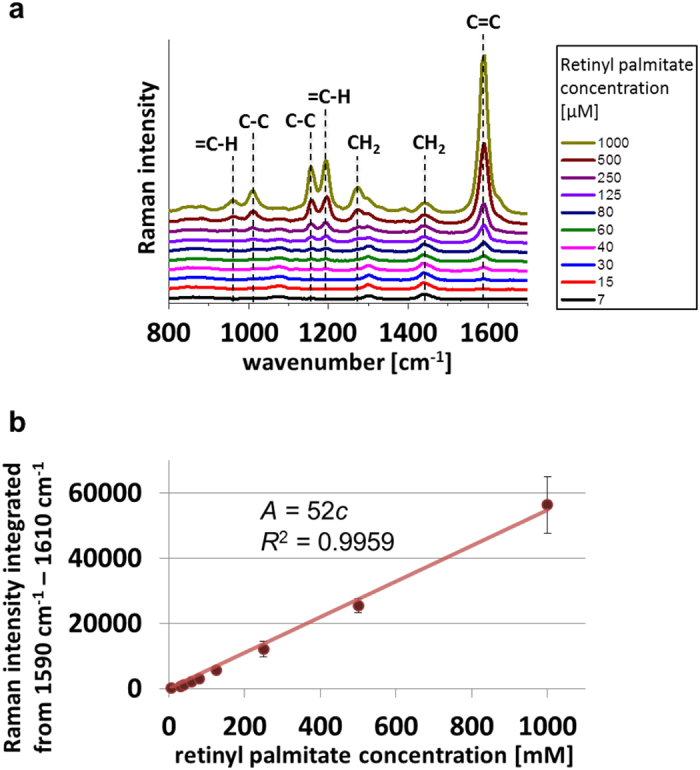
Retinyl palmitate in palmitic acid. (**a**) Raman spectra of different concentrations of retinyl palmitate in palmitic acid. Prominent bands are assigned. (**b**) Calibration curve visualizing the linear dependency of the integrated Raman intensity between 1590 cm^−1^ and 1610 cm^−1^ (*A*) from the retinyl palmitate concentration (**c**). Error bars indicate standard deviations of three independently prepared samples measured at three different days.

**Figure 2:**
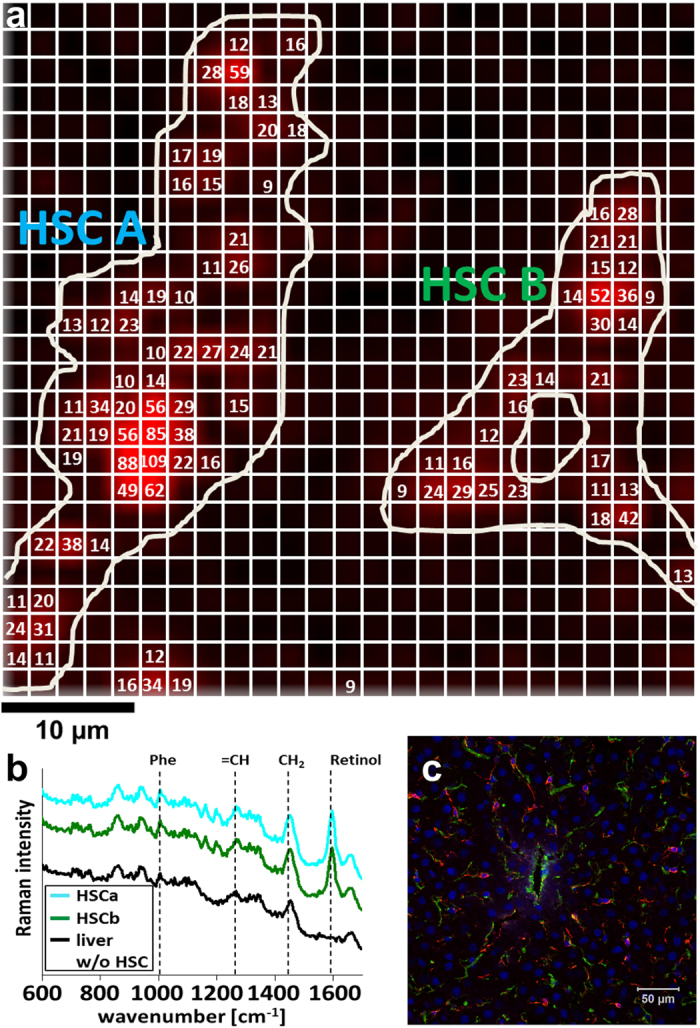
Visualization and quantification of the retinoid content of individual HSCs in liver tissue. (**a**) Raman false color image (from mouse 1) generated by the integration from 1590 cm^−1^ to 1610 cm^−1^ in each single spectrum. The white grating illustrates individual pixels of Raman mapping acquisition (step size 2 μm). The white line drawing sketches HSC positions, including nucleus position in HSC B. In addition, retinoid concentrations in mM calculated from the Raman spectra are specified as numbers in the respective pixels. (**b**) Average Raman spectra of the HSCs shown in (**a**) and of the surrounding tissue. Characteristic vibrational bands are assigned. The spectra are shifted along the ordinate axis for better visualization, the baseline is not corrected. (**c**) Fluorescence image of stained tissue from an untreated mouse visualizing HSCs (red, GFAP), blood vessel (green, collagen III) and nuclei (blue, DAPI). The scale bar indicates 50 μm.

In order to extract quantitative information from a Raman spectrum, reference information is essential. For spontaneous Raman scattering, the intensity I of the scattered Raman light is proportional to the number *N* of analyte molecules: *I* = *kN* with the constant of proportionality *k* containing molecule-specific parameters such as polarizability and experimental parameters such as excitation wavelength and intensity of the excitation light. For constant experimental conditions this relationship can be used to quantify analytes as was shown previously for intracellular molecules in cultured cells^7^ and the estimation of the limit of detection of heme and heme degradation products^8^. Here, calibration measurements were carried out with solutions of several quantities of retinyl palmitate (RP) in palmitic acid to correlate the scattering intensity of the C=C vibrational band and the retinoid concentration (Fig. 1a). RP had been chosen because it is the predominant retinol storage form in HSC lipid droplets. Palmitic acid was chosen as solvent because it provides excellent solubility for RP and also resembles the composition of the HSC lipid droplets. HSC lipid droplets are known to consist of retinyl esters, triglyceride, cholesterol, cholesteryl esters, phospholipids, free fatty acids and protein^9^. The reference spectra were recorded using the same measurement parameters as for the tissue experiments, except for temperature. Due to the freezing point of palmitic acid at 63 °C, a comparably high temperature of 75 °C was necessary for the reference measurements. For quantification, the integrated intensity of the main retinoid Raman band at 1593 cm^−1^ reflecting the C=C vibration was used (Fig. 1b). No vibrational contributions in the Raman spectra were specifically assignable to palmitic acid. Furthermore, the wavenumber region between 1590 cm^−1^ and 1610 cm^−1^ which was used for the calibration curve, is generally free of vibrational contributions from liver tissue (Fig. 2b).

### Retinoid content of HSCs in native liver

In order to assess the retinoid content of HSCs in a most physiological environment, a map of Raman spectra was recorded from a freshly sampled, unprocessed liver of a healthy mouse. It covered 50 μm by 50 μm with a step size of 2 μm. A false color Raman image reflecting the integrated intensities between 1590 cm^−1^ and 1610 cm^−1^ (Fig. 2a) indicates the presence of retinol. Retinol is stored as retinyl esters with fatty acids in lipid droplets in the HSCs^10^. Those droplets are distributed all over the cell^9^. Thus, bright color in the false color intensity image (Fig. 2a) indicates the HSC positions. Two HSCs were present in the scanned area.

**Figure 3:**
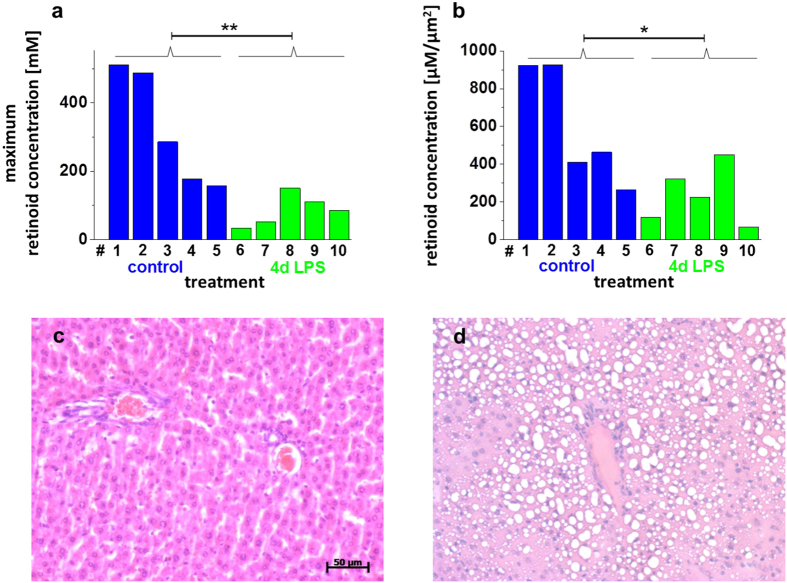
Assessment of murine liver tissue. (**a**,**b**) Illustration of the results on the hepatic retinoid state available from the Raman spectra of unprocessed liver sampled from 5 untreated (blue) and 5 LPS-treated (green) mice. The numbers on the x-axis indicate individual mice. The maximum retinoid concentration (**a**) is the global maximum found in 3–5 Raman maps per anmimal, the retinoid concentration per area (**b**) was computed for the whole scanned area (150–200 μm) per animal The average maximum retinoid content (**a**) differs significantly between untreated and LPS-treated mice (p = 0.004 < **) as does the average retinoid content per μm^2^ (**b**) (p = 0.03 < *). (**c**,**d**) Images of H&E-stained tissue representative for mice affected by the LPS treatment (**d**) as well as for untreated and mildly affected mice (**c**) (blue – nuclei, violet – cell cytoplasm, white circles in (**d)**-vesicles originally filled with lipids). The scale bar indicates 50 μm and is valid for both images (**c**,**d**).

Due to auto-fluorescence, raw Raman spectra of liver tissue suffer from a broad background (Fig. 2b). This background was subtracted prior to retinoid quantification. Furthermore, retinoid concentrations were calculated only if the scattering intensity at 1593 cm^−1^ in the pretreated spectrum surpassed a threshold of 100 CCD counts which corresponds to approximately five times the noise level in the spectra. A maximum local retinoid concentration of 109 mM within the focal volume of a scanned pixel was detected within HSC A (Fig. 2a). In HSC B a maximum concentration of 52 mM was detected. The maximum retinoid concentration of further cells detected in other Raman maps of in total 5 healthy, female mice (young adults) are presented together with the false color Raman maps in the Supplementary Information (Fig. S1). Overall maximum retinoid concentrations found in ten different animals are depicted in Fig. 3a.

The images shown in Fig. 2a and in the Supplementary Information denote Raman images visualizing the retinoid distribution in a native liver. The retinoids form droplet-like features. This is in agreement with current knowledge of the storage of retinoids in different lipid droplets within the cell: in small ones which are membrane surrounded (type I) and large ones without membrane (type II)^9^. The area filled with the retinoids in the Raman false color images closely resembles the typical shape of a HSC as can be revealed by immunofluorescence staining of the HSC-marker protein glial fibrillary acidic protein (GFAP) in tissue slices (Fig. 2c, Supplementary Information Fig. S2). Visualization of HSCs using their retinoid content has been achieved previously by Raman imaging from sections of frozen tissue^5^ as well as from retinoid autofluorescence from fixated and native liver tissue^9^,^11^. However, no quantification of the retinoid content has been demonstrated so far on the single cell level in native intact liver lobes. Thus, there exists no gold standard or reference value so far. A few bulk measurements have been performed determining the retinoid content within 10^6^ isolated HSCs^12^,^13^. In order to compare our data with those literature values we have to perform a few calculations as outlined in the next paragraph exemplarily for the two HSCs depicted in Fig. 2a.

Even though distributed almost evenly in the HSC, the different droplets showed different retinol concentrations in the Raman map (Fig. 2a). Thus, for further calculations, the average of the retinoid concentrations detected within each cell was computed and yielded average concentrations of 26 mM for HSC A and 20 mM for HSC B, respectively. Those values mark the lower concentration value within a particular cell as it cannot be guaranteed that the HSC fills the full focal volume and that the measured concentration in each pixel are the retinoid concentrations in the cell. As their name indicates, the HSCs possess many very thin, star-like protrusions that extend in all directions around the cell (Supplementary Information Fig. S2). It is very likely that with an imaging approach in one z-plane, which is collecting the signal from around 1.3 μm depth^14^, not only the HSC but also surrounding hepatocytes can contribute to the recorded signal. Thus, the retinoid signal will be “diluted” and the measured retinoid concentration is lower than the true retinoid content of the cell. Nevertheless, the obtained average values of the retinoid content can be used to estimate the total amount of retinoid in a HSC. For this estimation the molar weight of 524.86 g/mol of RP as major retinol storing form is used and the volume of a HSC is approximated with a spheroid with a diameter of 9 μm giving a volume of 382 fL. While HSCs in tissue are spread and occur in the typical stellate morphology (Fig. 2c, Supplementary Information Fig. S2), they adopt a spheroidal morphology once they are freshly isolated^2^ and as long as they do not adhere to a surface. 9 μm is the average diameter of a freshly isolated and agarose-embedded HSC from mouse. This value is also in agreement with size estimations from a z-stack of an immunofluorescently-labeled HSC in tissue (Supplementary Information Fig. S2): the diameter of the cell body is around 9 μm. Inclusion of the dimensions of the protrusions into the calculation gives an overall volume of around 435 fL. Further calculations are, however, based on the value determined for the isolated HSCs, namely 382 fL. If the cells were filled completely by lipid droplets containing RP, HSCs A and B possessed 5.2 pg RP and 4.0 pg RP, respectively. However, in HSCs the nucleus occupies about 20% of the cell volume^15^. Accordingly, a region poor in retinoids is visible centrally in HSC B in the false color image (Fig. 2a). Since the average retinoid concentration per HSC has been calculated only from those sites in the cell where the intensity around 1593 cm^−1^ in the corresponding Raman spectrum exceeded a threshold, the retinoid amount in the cell should be based on a reduced cell volume of 306 fL. Then the RP amount is 4.2 pg for HSC A and 3.2 pg for HSC B, respectively. Literature data derived from bulk measurements of the retinoid content of isolated HSCs by high pressure liquid chromatography suggest amounts between 100 μmol per 10^6^ HSCs from male, 8-weeks-old C57BL/6J mice^12^ and 3 nmol per 10^6^ HSCs from C57Bl/6N mice^13^, respectively. Accordingly, the average RP content per HSC would be 52 ng and 2 pg, respectively. This seems to be a quite wide range for an average value which we cannot explain with the available information and current knowledge. A single cell method as presented in this manuscript, which can quantify the retinoid content directly in the fresh liver tissue, could help to elucidate the effects of different factors on the retinoid concentration. It is known that the average retinoid content might vary remarkably with the nutrition (more or less rich in retinoids)^10^, however, no information on the diet is available from the two papers. Further differences might be caused by the sample treatment. The HSCs had been isolated using similar protocols for density gradient centrifugation in both studies. However, the average value might vary with the applied gradient. Generally, density gradient centrifugation can be expected to prefer HSCs containing many retinoids for isolation, because those HSCs containing only few retinoids will stay in another layer in the centrifugation tube together with other lighter cells. Nevertheless, the amounts calculated from the Raman data fit in the range measured by Radaeva *et al*.^13^. The fact that comparable values of the retinoid content were derived independently and by completely different approaches argues for the reliability of these results. In contrast to the bulk data, the Raman data account for inter-cellular differences and the anatomical localization of the HSC in the liver can be attributed. The latter is of special relevance since the retinoid content of HSCs was described to vary in dependence of the affiliation to a certain population and of their localization^16^,^17^.

### Variations in the hepatic retinoid state in a mouse model of systemic inflammation

Apart from that physiological variance, HSCs are known to lose retinoids in the course of activation occurring, e.g., in response to intoxication^3^. From the Raman spectroscopic analysis, information on the hepatic retinoid state becomes available in a minimal destructive manner. In order to assess its value to reflect the disease state, mouse livers sampled from healthy as well as lipopolysaccharide (LPS)-treated individuals were included for Raman spectroscopic investigation. LPS causes an inflammatory response in the organism which affects the liver^18^. Signs of severe hepatic steatosis were visible in the histological images of livers from two LPS-treated animals (Fig. 3d) while livers from animals of the control group did not show signs of hepatic steatosis (Fig. 3c). Signs of steatosis were very minor for three other LPS-treated animals. The maximum retinoid content and the retinoid content per μm^2^ were calculated from the Raman data for a small area in each liver and compared (Fig. 3a,b). Both values were significantly lower in liver samples from LPS-treated mice than in liver samples from the untreated controls (p = 0.004 regarding the difference in the maximum retinoid content, and p = 0.03 regarding the difference of the retinoid concentration per μm^2^). Of note, if hepatic injury was minor, as deducible from histology (three most right bars in Fig. 3a,b), the maximum retinoid content was constantly higher than in severe cases, although only few μm^2^ could be covered by the Raman assessment. Interestingly, HSCs are also known to experience an activation accompanied by a decrease in their retinoid content in the course of hepatic cirrhosis^3^,^19^. Thus, in that context, the height of the hepatic retinoid state might be useful as a marker for the severity of a hepatic insult or even for recovery. Via Raman spectroscopy, it can become accessible by light and without the necessity of liver destruction.

### Implications on hepatic health state from statistical analysis of the Raman spectra

**Figure 4:**
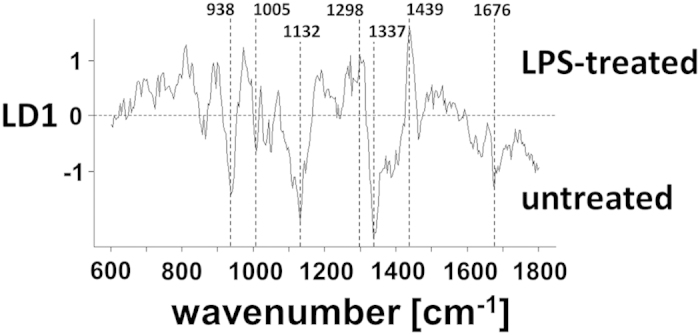
PCA-LDA coefficient depicting those vibrational contributions which dominantly affected the classification of a Raman spectrum in one of the classes “LPS-treated” or “untreated”. Important features are labeled.

Independent from the hepatic retinoid state, the Raman data derived from the fresh livers of the murine model of systemic inflammation point towards the possibility to address the general hepatic health state in an even more sensitive manner. For this purpose, the fingerprint region of the Raman spectra between 600 cm^−1^ and 1800 cm^−1^ was taken into account. A classification model based on principal component analysis (PCA) and linear discriminant analysis (LDA) was built to differentiate livers from LPS-treated mice (diseased) and untreated mice (healthy). It was possible to identify spectra of those LPS-livers unsuspicious in histology as belonging to a diseased liver with an accuracy of 81% (Table 1). In the LDA-coefficient visualizing the bands that are of relevance for the discrimination no contributions from retinoids are present (Fig. 4). This is in agreement with the retinoid state of the livers with minor signs of steatosis. Instead, features of proteins^20^ at 938 cm^−1^ (C-C), 1005 cm^−1^ (phenylalanine), 1132 cm^−1^ (C-N), 1337 cm^−1^ (CH) and 1676 cm^−1^ (amide I) were influential for the assignment into the class “untreated”. On the other hand, especially lipid vibrations^21^ at 1069 cm^−1^ (C-C), 1298 cm^−1^ (CH_2_) and 1439 cm^−1^ (CH_2_) caused a classification into the class “LPS-treated”. This is in accordance with the development of hepatic steatosis in response to LPS. Recently, the suitability of Raman spectroscopy for the characterization of hepatic steatosis was described for cryo-samples as well^22^.

## Conclusion

The Raman spectroscopy-based approach presented in this study allows for the assessment of the retinoid content of single HSCs in their native tissue surrounding. It does not only provide information on cell type identity, but rather gives access to the cell’s retinoid load which in turn reflects its state of activation. This will serve to characterize different HSC populations in the liver with regard to their retinoid content, potentially *in vivo,* in future. Due to the minor destructivity of Raman spectroscopy, samples stay available for assessments by other methods afterwards. Thus, the HSC retinoid state might be correlated with information on the expression of molecular markers. It might even itself become a clinical marker for hepatic health.

## Methods

### Animal experiments and liver sample preparation

All animal experiments were conducted in accordance with German legislation on protection of animals and with approval of the regional animal welfare committee of Thuringia. Female C57Bl/6 mice (Charles River, Germany) between 12 and 16 weeks of age were used for all experiments.

Primary HSCs embedded in agarose^2^ were used to derive the average diameter of a HSC. 23 cells were taken into account. The diameters of those cells ranged between 14 μm and 6 μm, the average was 9 μm.

Livers designated for Raman analysis were sampled freshly on the days of the measurements. Five mice belonged to the LPS treatment group, five mice belonged to the control group (no treatment). Mice in the LPS treatment group were injected once with 11 mg kg^−1^ body weight LPS into the peritoneum four days prior to sacrification. A section of each liver was cut, transferred into a cryo-vial and frozen in liquid nitrogen immediately. The remaining part was kept in physiological NaCl solution at 4 °C until it was analyzed by Raman spectroscopy. Samples for retinoid quantification in single HSCs were subjected to Raman spectroscopy within 1 h from sampling.

### Acquisition of Raman spectra

The Raman spectra were acquired with a Micro-Raman spectroscopy setup (alpha 300, Witec, Germany) employing a 785 nm diode laser for excitation and a deep-depletion back-illuminated charge-coupled device (CCD) (DU401A-BR-DD-352, Andor, Ireland) cooled to −60 °C for the detection of the Stokes-Raman signal. A 100× LD EC Epiplan Neofluar objective with NA 0.75 (Carl Zeiss AG, Germany) served to focus the laser light onto the sample and to collect the 180° backscattered light, respectively. The laser power before the objective was set to 100 mW. The backscattered light was led through a 100 μm fiber (acting as a large pinhole) and diffracted by a 300 lines per mm grating before hitting on the CCD detector. These settings allow for a theoretical resolution in x and y of


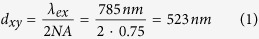


and in z of


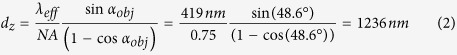


with


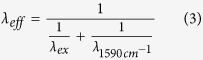


and


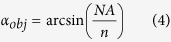


as outlined in detail by Heintzmann^14^.

Raman spectra from mixtures of retinyl palmitate and palmitic acid were recorded as series of 5 spectra with an integration time of 1 s per spectrum.

Raman spectra from in total 10 liver samples from 5 untreated mice and 5 LPS-treated mice, respectively, were recorded in mapping mode with 2 μm step size and 1 s acquisition time per spectrum. The relatively high step size was chosen to cover large tissue regions (up to 50 μm × 50 μm per map) while at the same time keeping the measurement time as low as possible (maximal 12 min/map) to avoid a change of focus during the measurement due to drying as well as to prevent possible photodamage to the retinoids. The mapping was performed by a continuous scan over the respective step size (2 μm) during the indicated integration time (1 s). Thus, the data represent an average (in x-direction) of the retinoid distribution in the respective pixel. Between 1475 and 1875 spectra (originating from 3 to 4 maps per sample) were acquired per liver sample.

### Retinoid calibration curve and retinoid quantification in the liver

The retinoid calibration curve was acquired with retinyl palmitate (RP) dissolved in palmitic acid in the concentration range between 7 mM and 1000 mM. Each concentration was prepared three times. From each sample five to seven series of five spectra were recorded. An average spectrum was calculated from each series. Those average Raman spectra were truncated to the wavenumber region between 1500 cm^−1^ and 1700 cm^−1^ and baseline corrected using second order polynomials. Afterwards, the intensities acquired between 1590 cm^−1^ and 1610 cm^−1^ were integrated for each spectrum. Replicate measurements (5 to 7) from the same sample were averaged. Values from three independent preparation times served to calculate average values and standard deviations per RP concentration depicted in Fig. 1b.

For retinoid quantification in the liver, raw spectra of the liver samples were truncated to the wavenumber region between 1500 cm^−1^ and 1700 cm^−1^ and spectra were baseline corrected with second order polynomials. If the intensity at 1593 cm^−1^ exceeded 100 CCD counts in the baseline corrected spectra, the corresponding retinoid concentration was calculated from the integrated intensity between 1590 cm^−1^ and 1610 cm^−1^ and with the help of the calibration curve.

### Principal component analysis-Linear discriminant analysis and significance calculation

In order to categorize the health state of a liver based on the Raman data of fresh tissue samples, the same spectra maps as for the retinoid quantification were used. This time, the wavenumber region between 600 cm^−1^ and 1800 cm^−1^ was kept from the raw spectra. Spectra were baseline corrected with second order polynomials and vector-normalized, spectra containing cosmic spikes were removed. Principal component analysis (PCA) was conducted to reduce the dimension of the data set. The first three principal components were used for subsequent linear discriminant analysis (LDA). For PCA-LDA, the data set was split in advance. The data of livers of three untreated animals and the data of livers of three LPS-treated animals (two severely affected and one mildly affected) were used to build the PCA-LDA model. Afterwards the spectra of the remaining livers were classified in one of the classes untreated or LPS-treated.

In order to test the statistical significance of the differences in the retinoid occurrence in livers of untreated and LPS-treated mice a Wilcoxon rank sum test, which is equivalent to the Mann-Whitney test, was carried out. This non-parametric test does not rely on the normality assumption of the data, thus can be applied to small sample sizes. The alternative hypothesis was that the control group features a higher maximal retinoid concentration and a higher averaged retinoid concentration, respectively. The threshold for statistical significance was set to be 0.05 (*), 0.01 (**) and 0.001 (***).

### Histological stains and image acquisition

4 μm slices were cut from the frozen sections with a cryo-microtome and subjected to hematoxylin and eosin staining or immunofluorescence staining as described previously^2^ to derive histological information on the hepatic condition. Glial fibrillary acidic protein served to identify HSCs, collagen III was used as the antigen to visualize blood vessel walls. A tissue section of about 50 μm was included for immunofluorescence staining to derive information on HSC extension in the tissue (Supplementary Information Fig. S2).

The immunofluorescence images were recorded with a laser scanning microscope (LSM 510 META, Carl Zeiss AG, Germany) using a 40× air objective (NA 0.75, Carl Zeiss AG, Germany) and a 63× oil immersion objective (NA 1.3, Carl Zeiss AG, Germany), respectively. The following lasers, beam splitters and filters were used for fluorescence excitation and emission detection of the different dyes: DAPI: laser diode 405 nm, HFT 405/514, BP 420–480; FITC: Argon laser 488 nm, HFT 488, NFT 490, BP 505–550; Cy3: HeNe laser 543 nm, HFT 405/488/543, NFT 490, BP 560–615.

The images of hematoxylin and eosin-stained tissue were acquired with a Zeiss Axio Vert.A1 microscope (Carl Zeiss AG, Germany) which was equipped with a LED as the illumination light source and a 20× objective (NA 0.3, Carl Zeiss AG, Germany).

## Additional Information

**How to cite this article**: Galler, K. *et al*. Single cell analysis in native tissue: Quantification of the retinoid content of hepatic stellate cells. *Sci. Rep.* **6**, 24155; doi: 10.1038/srep24155 (2016).

## Supplementary Material

Supplementary Information

## Figures and Tables

**Table 1:** PCA-LDA results.

		**Prediction**	
		**Untreated (healthy)**	**LPS-treated (diseased)**
reference	untreated	3036	506
	LPS-treated	782	2545

Spectra recorded from two untreated and two LPS-treated (both mildly affected) mouse livers were classified as untreated (healthy) or LPS-treated (diseased) by PCA-LDA. The accuracy is 81%, with a sensitivity of 76% and a specificity of 86%. The PCA-LDA model had been built by biologically and statistically independent data of livers of three untreated and three LPS-treated (two severely affected, one mildly affected) mice. See the coefficient corresponding to that model in Fig. 4.
