# Supplementary material for: Single cell analysis in native tissue: Quantification of the retinoid content of hepatic stellate cells
Source: Sci Rep. 2016 Apr 11;6:24155. doi: 10.1038/srep24155 (PMC4827054; doi:10.1038/srep24155)
Supplement: Supplementary Information [file srep24155-s1.pdf]

Supplementary Figures

Single cell analysis in native tissue: Quantification of the retinoid content of hepatic stellate cells

K. Galler[a,b], R. P. Requardt[b], U. Glaser[a,b], R. Markwart[b], T. Bocklitz[4], M. Bauer[b,c], J. Popp[a,b,d, e], U. Neugebauer[a,b,e]

[a] Leibniz Institute of Photonic Technology, Jena, Germany, [b] Center for Sepsis Control and Care, Jena University Hospital, Germany, [c] Department of Anesthesiology and Intensive Care Medicine, Jena University Hospital, Germany, [d] Institute of Physical Chemistry and Abbe Center of Photonics, Friedrich Schiller University Jena, Germany, [e] InfectoGnostics Research Campus Jena, Center for Applied Research, Jena, Germany

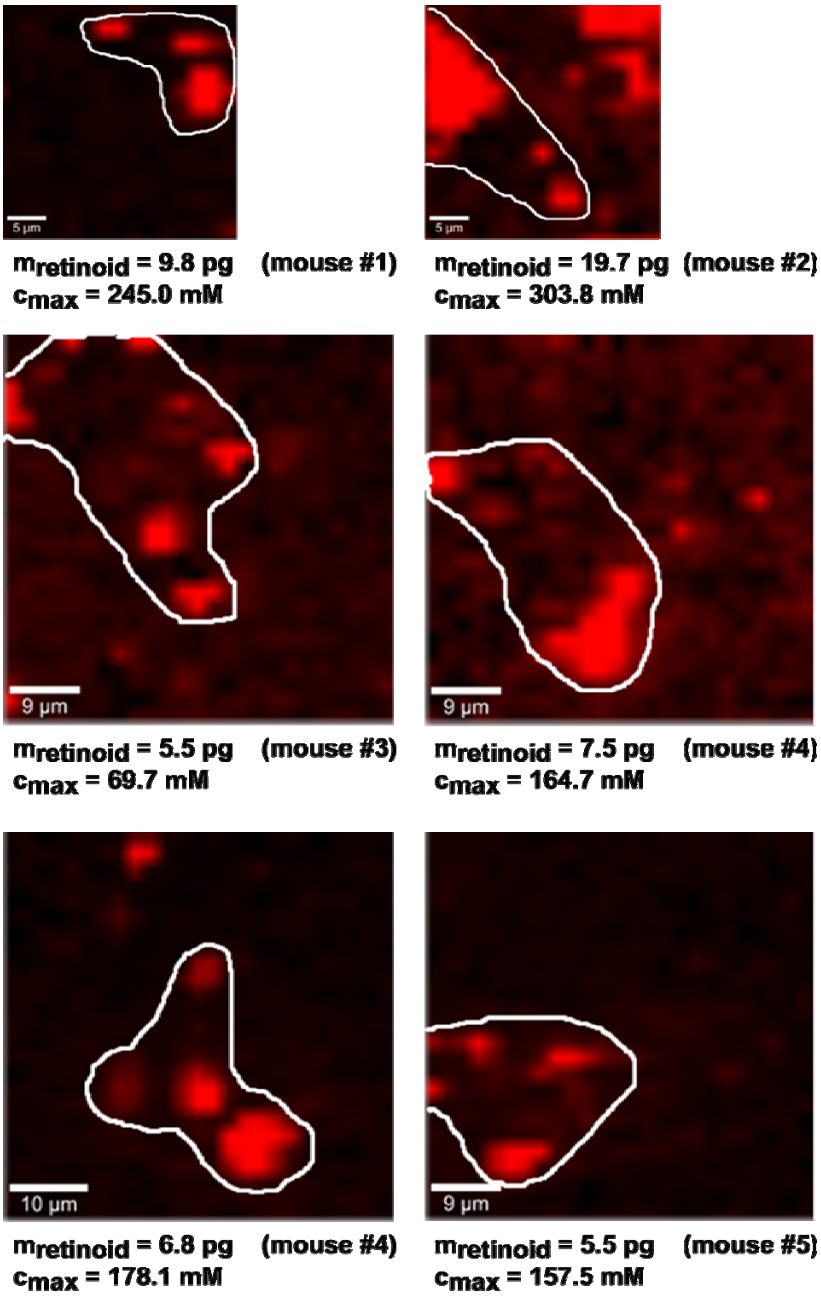

Supplementary Figure S1: Hepatic stellate cells (HSCs) in native livers of healthy mice. Values below each image denote the total content of retinoids and the maximum concentration of retinoids derived as described for the cells in Figure 2 a in the main paper. The mouse numbers correspond with those numbers used in Figure 3 a and b in the main paper. The white frame sketches what is deemed to be an individual HSC.

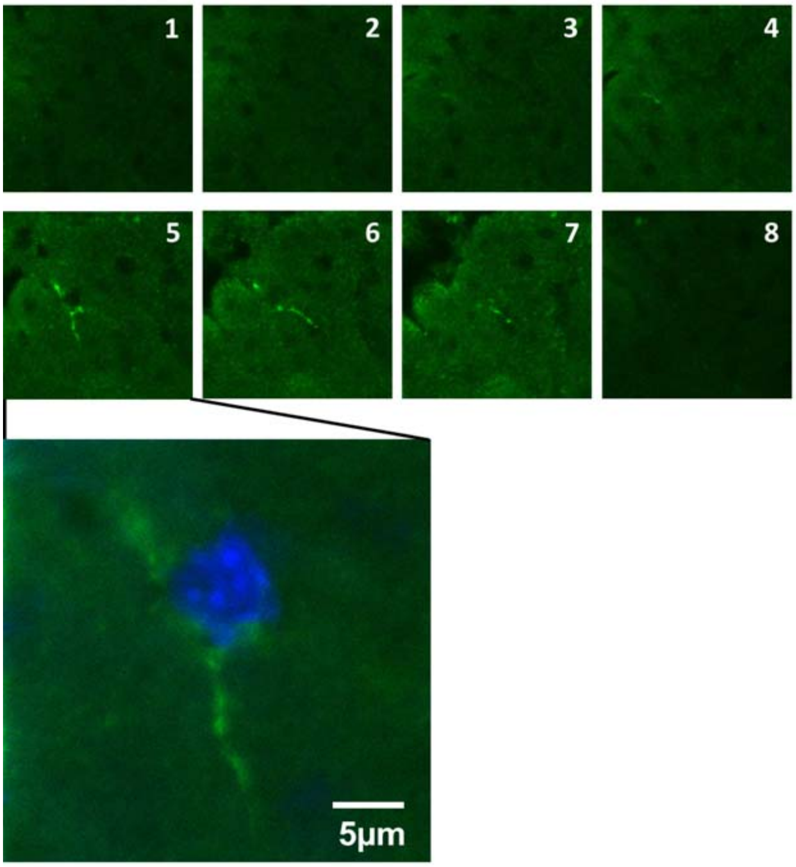

Supplementary Figure S2: Hepatic stellate cell in liver tissue. Fluorescence images from a z-stack of immunofluorescently-labeled liver tissue are depicted. Glial fibrillary acidic protein (green) marks the HSC, the nucleus was stained with DAPI (blue). The step size in z-direction was 5 µm. Thin protrusions of one HSC are visible in different layers of the stack. The unspecific green background results from unspecific retention of antibodies in the thick sample.
